# Thermochemical behavior of agricultural and industrial sugarcane residues for bioenergy applications

**DOI:** 10.1080/21655979.2023.2283264

**Published:** 2023-11-20

**Authors:** Karen Lorena Martinez-Mendoza, Juan Guerrero-Perez, Juan Barraza-Burgos, Carmen Rosa Forero, Orla Williams, Edward Lester, Nicolas Gil

**Affiliations:** a Facultad de Ingeniería, Universidad del Valle, Ciudad Universitaria Meléndez, Cali, Colombia; b Faculty of Engineering, University of Nottingham, University Park, UK; c Cenicaña, Colombian Sugar Cane Research Center, Cali, Colombia

**Keywords:** Biomass combustion, sugarcane residues, thermogravimetric analysis, bioenergy, renewable fuels

## Abstract

The Colombian sugarcane industry yields significant residues, categorized as agricultural and industrial. While bagasse, a widely studied industrial residue, is employed for energy recovery through combustion, agricultural residues are often left in fields. This study assesses the combustion behavior of these residues in typical collection scenarios. Additionally, it encompasses the characterization of residues from genetically modified sugarcane varieties in Colombia, potentially exhibiting distinct properties not previously documented. Non-isothermal thermogravimetrical analysis was employed to study the thermal behavior of sugarcane industrial residues (bagasse and pith) alongside agricultural residues from two different sugarcane varieties. This facilitated the determination of combustion reactivity through characteristic combustion process temperatures and technical parameters like ignition and combustion indexes. Proximate, elemental, and biochemical analyses revealed slight compositional differences. Agricultural residues demonstrated higher ash content (up to 34%) due to foreign matter adhering during harvesting, as well as soil and mud attachment during collection. Lignin content also varied, being lower for bagasse and pith, attributed to the juice extraction and milling processes that remove soluble lignin. Thermogravimetric analysis unveiled a two-stage burning process in all samples: devolatilization and char formation (~170°C), followed by char combustion (~310°C). Characteristic temperatures displayed subtle differences, with agricultural residues exhibiting lower temperatures and decomposition rates, resulting in reduced ignition and combustion indexes. This indicates heightened combustion reactivity in industrial residues, attributed to their elevated oxygen percentage, leading to more reactive functional groups and greater combustion stability compared to agricultural residues. This information is pertinent for optimizing sugarcane residues utilization in energy applications.

## Research Highlights

- Weather in collection time affects composition of sugarcane agricultural residues.
- Combustion of sugarcane residues occurs over similar temperature ranges.
- Industrial residues are more reactive to combustion than agricultural residues.
- Overall thermal behavior of sugarcane residues depends on their composition.

## 1. Introduction

The pressing global concern over CO_2_ emissions from fossil fuels has spurred a surge in the adoption of renewable and sustainable fuels worldwide [1]. Among these alternatives, biomass stands out as a frontrunner in the battle against global warming, operating within a CO_2_ fixation and release cycle [2]. Recognized as a pivotal renewable energy resource, biomass plays an increasingly vital role in climate protection efforts [3]. Colombia, for example, annually generates a substantial 71 million tons of agricultural residues [4], showcasing significant potential for bioenergy production. This includes over 5 million tons of sugar cane bagasse (the fibrous residue left after extracting juice from sugar cane in the sugar production process), 457,000 tons of rice straw, and a total of 29 million tons of residual agricultural biomass, all ripe for harnessing in bioenergy applications [5]. In the Valle del Cauca region, a prominent sugarcane-growing area, the residues from sugarcane harvesting, known as Green Harvesting Residues (GHR), represent a significant resource. GHR encompasses green leaves, dried leaves, and buds, which are commonly left in the fields after sugarcane harvesting. It yields approximately 1.82 million tons annually, with a calorific value of 16,965 kJ/kg [6]. The shift from manual to mechanized sugarcane harvesting has led to an abundance of residues, necessitating efficient disposal methods, as burning is no longer a viable option [7].

Coal-fired power generation currently constitutes 8.9% of Colombia’s electricity production [8]. In Europe, the conversion of biomass in coal-fired power stations has yielded significant reductions in carbon emissions [9]. Repurposing coal plants for biomass utilization presents a substantial opportunity to markedly reduce Colombia’s carbon footprint. In 2017, Colombian biomass-based electricity reached 793 MWh, primarily sourced from sugar mills burning bagasse [5]. The deployment of low-pressure boilers in sugar mills for electricity, steam production, and residue management is a common practice [10]. Notably, in 2020, cogeneration with bagasse from sugar mills in specific regions accounted for 99.8% of Colombian biomass energy production [11]. By utilizing agricultural residues for combustion, the sugarcane industry can address the issue of green harvesting residues left in the fields while simultaneously reducing carbon emissions from electricity generation.

Currently, in addition to direct combustion as a strategy for exploiting these residues, there are innovative applications of sugarcane waste, such as anaerobic production of hydrogen from bagasse [12], biogas production [13], and even the extraction of materials extensively required by the modern industry like silica [14]. These demonstrate the diverse potential of using these residues in modern technologies. Additionally, strategies like pyrolysis and gasification have been investigated [15], which, along with combustion, aim to harness the energy potential of these residues. However, the energy utilization of biomass, especially from agricultural residues like GHR, poses challenges due to its sensitivity to initial composition and weather conditions during collection. Limited studies exist on GHR combustion [16–21], also known as sugarcane leaves or straw in some cases. It is worth noting that these studies may not be universally applicable to all sugarcane varieties, given the genetic modifications undertaken based on specific soil conditions and production goals.

This study primarily aims to evaluate the thermal behavior of sugarcane agricultural residues, specifically Green Harvesting Residues (GHR), using non-isothermal Thermogravimetric Analysis (TGA). The focus lies in discerning differences between these agricultural residues and industrial residues like sugarcane bagasse (SB) and sugarcane pith (SP). These distinctions will be assessed in terms of proximate, elemental, and biochemical composition and their impact on thermochemical behavior during combustion. The GHR samples were collected during two predominant Colombian weather conditions, namely the rainy and dry seasons. These variations in weather significantly influence the biochemical composition of the samples, resulting in distinctive characteristics that differentiate agricultural residues from industrial residues such as bagasse. The research endeavors to offer crucial insights into the thermal behavior of different sugarcane residue samples and underscores the importance of ascertaining whether combustion can be integrated into the utilization of agricultural residues to the same extent it has been integrated into the use of industrial residues.

## 2. Materials and methods

### 2.1. Sample collection and preparation

Two samples of sugarcane green harvesting residues (mainly leaves, shoots, and short stems), GHR1 and GHR2, were collected from crop fields, while sugarcane industrial residues, bagasse (SB), and pith (SP), were obtained from a sugar mill in the Valle del Cauca region, southwest Colombia. GHR1 was collected during the dry season, and GHR2 was collected during the rainy season, both originating from indigenous sugarcane varieties developed by Cenicaña, the Colombian Sugarcane Research Center. The collection of GHR samples involved gathering residues left in the field after mechanical sugarcane harvesting, with GHR1 collected before tedding and GHR2 collected after mechanical tedding of the residues.

Subsequently, the samples were dried with atmospheric air until a constant weight was achieved. They were then quartered using a 12-riffle splitter to obtain representative samples. Finally, GHR, SB, and SP samples were milled using a Retsch ZM200 knife mill with a 4 mm screen. All samples were stored in sealed bags until further use.

### 2.2. Biomass characterization

Samples were reduced to <250 µm for proximate and elemental analysis using knives mill with a 0.1 mm screen. Moisture, volatile matter, ash, and fixed carbon were obtained according to ISO 18,122–2015 (ash) [22], 18123–2015 (volatile matter) [23], 18134-2-2015 (moisture) [24], and the fixed carbon (percent dry basis) is the difference between 100 and the sum of the ash and volatile matter percentage yields, determined on a dry basis. High heating value (HHV) determination was made in a calorimetric pump IKAC5000 using 1.00–1.50 g pellets of 13 mm diameter (Mold SPECAC 13 mm), which was calibrated using benzoic acid from manufacturer, according to the standard test method ASTM D5865–13 [25], originally developed for coal. Elemental composition was carried out according to ISO 16,996–2015 [26] in a Leco CHN628 and Sulfur 628S. The analysis of polymeric composites was performed according to the methodology described by Ayeni et al. [27] for GHR1, and the calculations of Debiagi et al. [28] for GHR2, SB, and SP.

### 2.3. Thermogravimetric analysis

Thermal behavior of coal and biomass can be studied by thermogravimetrical analysis TGA [29]. Information provided by TGA can be used to preliminarily assess combustion at large scale [30]

TGA can be isothermal or dynamic (non-isothermal). Isothermal TGA is used to determine the thermal stability of the sample, decomposition rate, and gas effect over decomposition rates (such as adsorption-desorption reactions) and kinetic of decomposition reactions. On the other hand, non-isothermal TGA relates weight loss with temperature in oxidative atmosphere.

Thermal behavior was studied using a non-isothermal micro-TGA TA500 in a dried-air atmosphere using a single heating ramp of 10°C/min until 900°C. Combustion reactivity was determined using the maximum mass combustion rate $R_{max},$average mass combustion rate $R_{av},$ and the characteristic temperatures: Ignition temperature (T_i_), peak temperature (T_p_) and burnout temperature (T_b_) obtained from differential thermograms. Ignition index D and the comprehensive combustion index S were calculated according to Equation (1) and Equation (2), respectively [31–33]. Both indexes increase with higher mass combustion rates and lower characteristic temperatures, therefore an increase in S and D is referred to faster ignition and combustion at lower temperatures.

(1) $$D=\frac{R_{max}}{T_{p}T_{i}}$$

(2) $$S=\frac{R_{max}R_{av}}{T_{i}^{2}*T_{b}}$$

## 3. Results and discussion

In this study, the thermal behavior of industrial and agricultural sugarcane residues was evaluated. The research presents the physicochemical characterization of the residues and the combustion profiles as a starting point to establish differences and similarities in their thermal behavior. This approach allows for an assessment of whether their combustion exhibits distinctive features or shares significant similarities. The aim is to promote the utilization of agricultural residues in line with the established practice for industrial residues.

### 3.1. Proximate and ultimate analysis

In Table 1, a proximate analysis is presented for GHR1, GHR2, SB, and SP. It is evident that GHR2 exhibited the highest ash content. This can be attributed to the rainy conditions during sample collection, as the increased humidity facilitated the adhesion of mud and soil to the GHR surface, which was subsequently collected. The lowest ash content was observed in the bagasse, as it is a residue that undergoes industrial processing before use. Furthermore, the ash contents of GHR1 and bagasse are similar. In general, the fixed carbon content is low for GHR, reaching a maximum of 9.2% for GHR1. Nevertheless, the fixed carbon content of GHR is higher than that of SB and SP, indicating slower combustion. Residues with higher fixed carbon content are also associated with higher activation energy, although this is contingent on the distribution of polymeric compounds such as cellulose and hemicellulose [34]. Regarding high heating value, all analyzed samples were found to be within the same order of magnitude, with minor variations among them. The lowest high heating value coincided with sample GHR2, whose ash concentration was the highest (34.1%).

**Table 1.** Biomass proximate analysis, %w/w (db)*.

Biomass	Moisture %	Volatile matter, %	Ash, %	Fixed carbon, %**	HHV, MJ/Kg
GHR1	6.8 ± 0.3	73.7 ± 0.5	17.1 ± 0.6	9.2 ± 0.3	16.555 ± 0.040
GHR2	6.3 ± 0.0	58.3 ± 0.4	34.1 ± 0.0	7.6 ± 0.4	16.344 ± 0.046
SB	7.5 ± 0.3	78.7 ± 0.1	15.0 ± 0.5	6.3 ± 0.6	16.727 ± 0.040
SP	8.2 ± 0.1	86.1 ± 0.4	9.6 ± 0.1	4.3 ± 0.5	16.425 ± 0.040

* Dry basis, **Calculated by difference.

The proximate analysis presented in Table 1 aligns with findings from other studies that have compared the characterization of industrial and agricultural residues from sugarcane. These studies consistently indicate that industrial residues like bagasse tend to have higher volatile content, resulting in a lower proportion of fixed carbon, leading to an enhanced reactivity in combustion processes. Additionally, they highlight a higher ash content in agricultural residues, referred to as straw or trash in these studies, mentioning the challenges this represents for the integration of agricultural residues into combustion systems, particularly with regard to the slagging and fouling problems [35,36].

In general, it is observed that GHR1 composition presents values very close to those measured for bagasse, so that this residue could be considered as a substitute for the bagasse currently used in co-firing with coal, without incurring a drastic change in the characteristics of the raw material. The main drawback for agricultural residues utilization is the high ash content. In Colombia, ash content in the boilers can be up to 30% so GHR1 ash content would not cause operational issues. GHR1 corresponds to the residues collected during the dry season before tedding so as a first result, only GHR1 could be used for energy production without further treatment. On the other hand, for international energy purposes, according to the ISO 17,225:2014 standard [37], herbaceous biomass intended for biofuel pellets should have an ash content below 10%. In this regard, only SP meets this criterion for biofuel pellet production.

**Figure 1.**
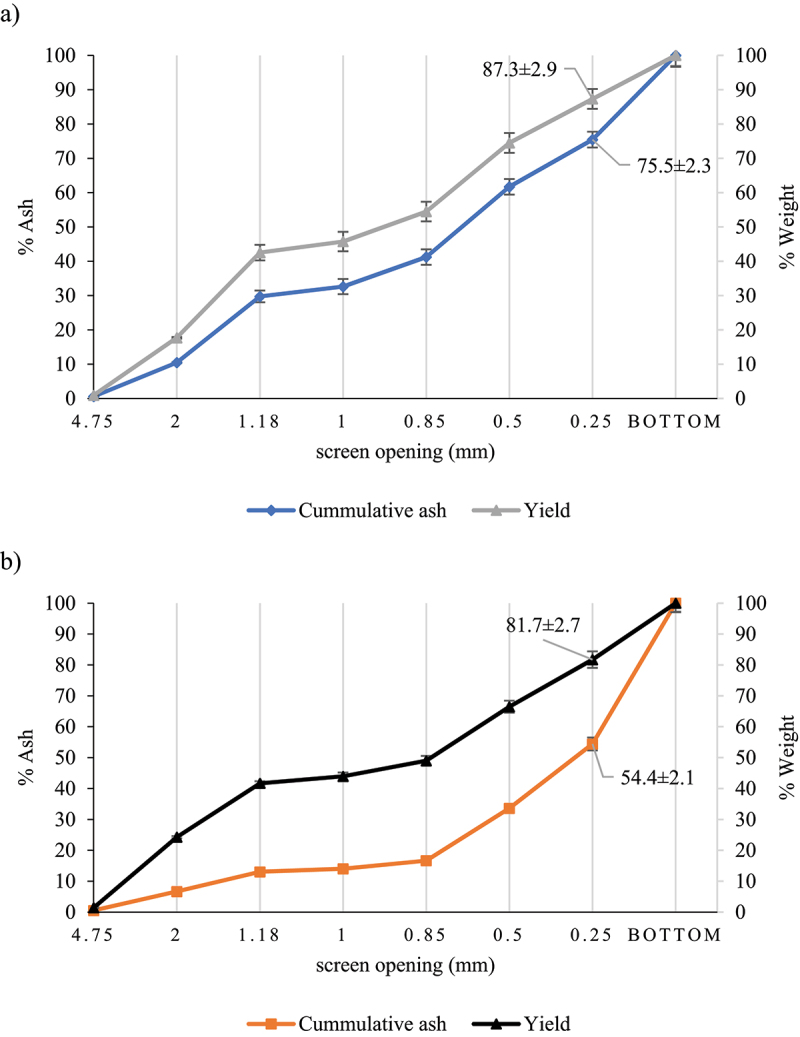
Cumulative ash and weight percentage for a) GHR1 and b) GHR2.

Nonetheless, there are several techniques that could be evaluated to decrease the ash content of the agricultural residues collected during the rainy season, like GHR2. These techniques may involve processes such as washing, leaching, sedimentation, and fractionation, which selectively remove a particular high ash fraction of the biomass, improving its suitability for energy production and other applications [38–40]. To address the high ash content in agricultural residues, this study analyzed the distribution of ash in different particle sizes. Figure 1 illustrates the cumulative ash content and cumulative weight distribution for samples GHR1 and GHR2. By removing particles smaller than 0.250 mm, the ash content of GHR1 decreased from 17% to 10% with an 87.3% yield. Similarly, for GHR2, the ash content decreased from 34.1% to 12.0% with an 81.7% yield. This represents a significant reduction of over 40% in ash content for the GHR collected during the rainy season.

In Table 2, elemental analysis is shown for GHR1, GHR2, SB, and SP. The carbon content was similar for all analyzed biomass samples, at around 35–40%. Coal carbon content is usually around 60–80%, which is related to higher carbonization processes compared with biomass [41]. Moreover, carbon content is directly related to higher heating value as it is normally used as a variable for HHV calculations from elemental analysis [42]. Hydrogen and oxygen contents are related to devolatilization behavior. In primary devolatilization, the biomass releases preferably oxygen and hydrogen by oxidation and hydrogenation reactions [43]. Since all samples have a similar hydrogen content (around 5%), it is likely that the release of volatile matter is consistent among all residues with respect to hydrogenation reactions. In terms of oxygen content, it is higher for SB and SP compared with GHR samples, which indicates a higher reactivity for SB and SP since superficial oxygen compounds promote combustion by reducing stoichiometric oxygen and therefore, accelerating the process by skipping the oxygen adsorption on the surface, which is the slowest stage in combustion [44,45]. Finally, nitrogen and sulfur content are similar for all studied biomass samples at < 1%.

**Table 2.** Biomass ultimate analysis, %w/w, db×.

Biomass	C	H	N	S	O**	Ash
GHR1	39.9±0.2	5.7±0.1	0.6±0.0	0.31±0.03	36.5±0.8	17.0±0.6
GHR2	37.9±0.7	5.4±0.1	0.7±0.0	0.17±0.01	21.7±0.8	34.1±0.0
SB	32.3±0.7	4.7±0.1	0.5±0.0	0.05±0.01	47.5±1.3	15.0±0.5
SP	41.3±0.1	5.9±0.1	0.7±0.1	0.04±0.01	42.4±0.4	9.6±0.1

*Dry basis, **Calculated.

**Figure 2.**
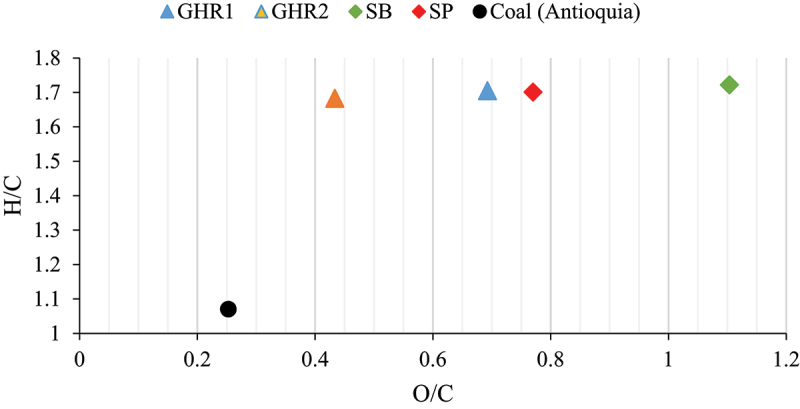
Van Krevelen diagram of the biomass samples.

Molar relations H/C and O/C are indicators of the type of bonding in biomass structure and can be related with the type of polymeric compounds present. C-H and C-O bonds release a smaller amount of energy when broken than C-C bonds, so the heating value increases as the H/C and O/C ratios decrease [46]. In addition, since breaking C-H and C-O bonds requires less energy than breaking C-C bonds, the higher the H/C and O/C ratios, the higher the reactivity of the fuel. Van Krevelen diagram is displayed in Figure 2. It is observed that all the studied biomasses exhibit a similar atomic H/C ratio. This suggests that during the devolatilization stage, similar behaviors can be anticipated for all the samples. On the other hand, while the hydrogen content is generally in the range of 5% for most coals [47] – similar to the residues under study – the higher carbon content in coal results in lower H/C ratios. This is exemplified in Figure 2, where a bituminous coal (from Antioquia-Colombia) is provided as a reference for comparative purposes. This distinction underscores a greater presence of C-H bonds in biomass compared to coal.

Industrial residues SB and SP presented the highest values of atomic O/C ratio, followed by GHRs samples. Therefore, it would be expected that sugarcane industrial residues have a higher reactivity than the GHRs. Referenced coal, on the other hand, has lower atomic H/C and O/C ratios, which makes it less reactive but has a higher heating value when subjected to combustion.

Other authors have concurred with a similar analysis, demonstrating that biomass generally exhibits H/C and O/C ratios that can be between two to four times higher than those of coal, depending on the rank or degree of carbonization of the coal. This explains the low calorific value of biomass, but also its high reactivity in primary combustion reactions involving both hydrogen and oxygen, compared to coal [48].

### 3.2. Biochemical composition

An experimental biochemical composition analysis was performed for GHR1, classifying the sample according to the part of the plant where it comes from. Shoots, stems, and leaves were analyzed by acid hydrolysis; this method is only suitable for biomass with low ash content (<10%); therefore, it could not be used for GHR2. This ash content limitation applies for most of the biochemical composition methods [49,50]. The biochemical composition, reported on a dry ash-free basis, is presented in Table 3.

**Table 3.** GHR1 biochemical composition %(dafb)*.

				Lignin	
GHR1	Extractives	Hemicellulose	Cellulose	Soluble	Insoluble
Leaves	15.70±0.05	34.93±0.38	38.72±1.51	2.90±0.08	7.75±1.00
Stems	17.77±0.05	28.92±1.05	40.00±1.47	2.87±0.07	10.44±0.65
Shoots	15.45±0.05	36.65±1.09	39.37±1.48	3.15±0.13	5.35±0.21

*Dry ash-free basis.

Similar to the findings of Debiagi et al. [28], GHR1 demonstrates a high extractive content, exceeding 15%, a trait shared with herbaceous biomass like switchgrass, which exhibited a content of 16.99%. The lignocellulosic composition varies depending on the part of the sugarcane plant analyzed. Leaves and shoots are more similar to each other, while stems have higher lignin content and lower hemicellulose. Lignin, being the strongest biochemical compound, provides attachment for the cellulose/hemicellulose fibers of the plants. Therefore, it is more prevalent in the structurally robust parts of the plant [46]. Hemicellulose, an amorphous component, exhibits variable composition depending on the type of biomass, usually falling within the range of 30–40% content. For stems, the lower hemicellulose value indicates lower reactivity compared to leaves and shoots.

The biochemical composition of GHR1 indicates that the majority of the energy derived from GHR comes from the thermochemical decomposition of hemicellulose and cellulose. As noted by Rego et al. [51], lignocellulosic biomass tends to decompose as a mixture of its biochemical compounds. Therefore, it is expected to observe peaks of decomposition around 300°C and 350°C, which are the typical peak decomposition temperatures for hemicellulose and cellulose, respectively [52]. The biochemical composition of the studied biomasses, including GHR samples (GHR1 and GHR2), SB, and SP, was determined using the mathematical algorithm developed by Debiagi et al. [28]. The method consists of an algorithm that employs three theoretical mixtures of cellulose, hemicellulose, and lignin, each with their respective elemental composition of carbon, hydrogen, and oxygen. The resulting polymeric composition of the biomasses is then calculated as a linear combination of these theoretical mixtures, based on the elemental analysis presented in Table 2.

Considering that for GHR1 elemental analysis was performed on leaves (main constituent of collected GHR ~ 99%) a comparison between experimental and mathematical biochemical composition determination is shown in Table 4. A maximum 10.7% relative error was determined for lignin and a minimum 1.7% for hemicellulose. A thermal method such as the one described by Cano Díaz et al. [53], could be explored for experimental biochemical composition determination of agricultural residues with high ash content.

**Table 4.** Biochemical composition determination comparison for GHR1 (leaves).

Compound	Mathematical method	Experimental method	Error relative
Cellulose	41.9	38.7	8.3
Hemicellulose	34.3	34.9	1.7
Lignin	9.5	10.7	10.6
Extractives	14.3	15.7	9.1

Biochemical composition for GHR, SB, and SP is very similar among them as shown in Table 5. Highest lignin content was obtained for GHR1 and GHR2, and highest hemicellulose/cellulose content was obtained for industrial residues SB and SP. These results are attributed to the additional juice extraction and milling processes, which remove soluble lignin from industrial residues [54]. Similar findings have been reported by other authors when comparing the biochemical composition of these two types of sugarcane residues [55]. These differences are correlated with variances in plant tissues and their processing history. This explains the lower lignin content in the bagasse, as it undergoes an extraction process, specifically warm water extraction, which is not the case for the straw.

**Table 5.** GHR1, GHR2, SB, and SP biochemical composition, %w/w, db×.

	Cellulose	Hemicellulose	Lignin	Extractives
GHR1	41.9	34.3	9.5	14.3
GHR2	41.6	34.1	9.7	14.6
SB	44.4	36.3	7.7	11.6
SP	46.1	37.7	6.5	9.7

*Dry basis.

### 3.3. Adiabatic flame temperature

The adiabatic flame temperature refers to the maximum temperature that a fuel would reach if there were no energy losses in the form of heat dissipation or work. It is especially important in combustion processes since, if the adiabatic temperature is higher than the ash fusibility temperature, there is a probability that the ash will melt, causing slagging or fouling problems, which are considered serious problems in the operation of a boiler [32].

An estimate based on simplifications of the complete energy balance of the combustion process was used to calculate the adiabatic flame temperature using Equation (3). $T_{in}$ is the initial temperature in K, LHV is the lower heating value in J/Kg, f is the mass ratio of fuel/air and C_p,gas_ refers to the calorific value for produced gas in J/(Kg·K). In this work, the calorific value of the gas was taken from nitrogen calorific value.

(3) $$T_{adiab}=T_{in}+\frac{LHV*f}{C_{p,gas}}$$

Table 6 shows that highest adiabatic temperatures are calculated for SB. Bagasse has the highest H/C and O/C relations, then less stoichiometric oxygen is required in combustion and therefore, less nitrogen is added into the fuel/air mix. Nitrogen acts as an inert gas in the combustion reaction, absorbing energy from the reaction. In general, for all biomass samples, adiabatic flame temperatures are around 2000°C. Lowest temperatures were found for GHR samples, for which LHV is the lowest (14402 KJ/Kg).

**Table 6.** Calculated adiabatic temperatures (°C) for GHR, SB, and SP.

Sample	No air excess	50% air excess	100% air excess	200% air excess
GHR1	2093	1583	1224	846
GHR2	1577	1181	910	627
SB	3091	2386	1865	1301
SP	2244	1703	1319	913

Ballesteros et al. [56] calculated an adiabatic flame temperature of 772°C using 50% of excess air with sugarcane leaves. In the reported data, a higher calorific value (15600 KJ/kg), lower ash content (3.85%), higher carbon (42.94%), hydrogen (6.26%), and oxygen content (46.65%) are observed in comparison with the data reported in this work. Higher carbon and hydrogen content increase the stoichiometric oxygen required and therefore the stoichiometric air required; so then, the amount of nitrogen present in the flue gas also increases, achieving a lower adiabatic flame temperature. Cobo Barrera [57], calculated the adiabatic flame temperature for agricultural cutting residues at 944°C (1218 K). In this case, the discrepancies in the results are due to the differences between the upper and lower calorific value used, since the author reports a lower calorific value of 3.36 MJ/kg for the residues, while in this work the values are around 15 MJ/kg. Meanwhile, Toscano Morales & Barriga [58] calculated an adiabatic flame temperature of 1215°C for bagasse. The differences in this case are due to differences in the lower heating value used; although the upper heating value reported is similar, for the calculation of the lower heating value, the authors used bagasse with 50% moisture. This value influences the equation for calculating the adiabatic flame temperature by decreasing the energy released in combustion by 50%, so the temperature values decrease drastically compared to the adiabatic temperature obtained in this work.

### 3.4. Thermal analysis of fuel samples

**Figure 3.**
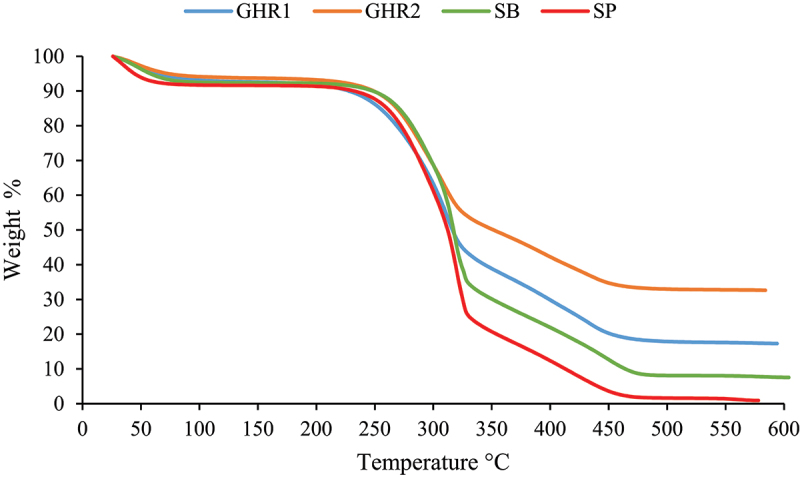
Sugarcane residues TG diagram at 10°C/min.

**Figure 4.**
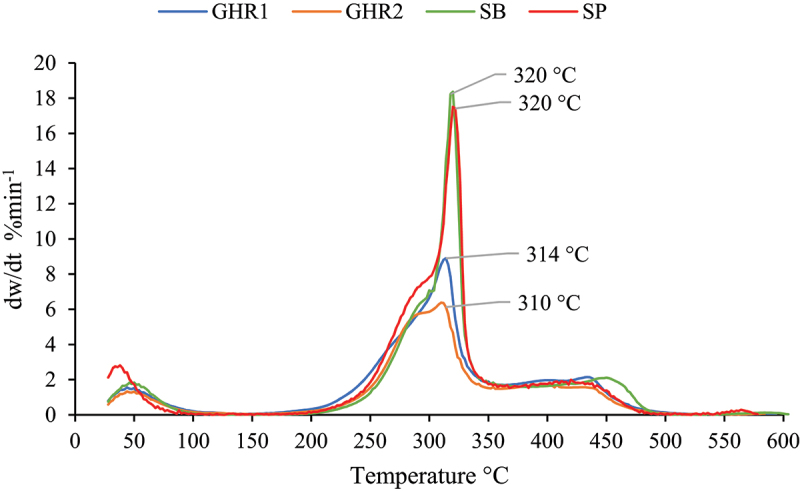
Sugarcane residues DTG at 10°C/min.

It was observed from the TGA thermograms in Figure 3 and the DTG curves in Figure 4 that a small weight loss occurs at temperatures between 25 and 105°C for all biomass samples, which is associated with moisture evaporation. The TGA diagrams exhibit similarity across all residues. However, around 310°C, although the thermograms maintain their shape, the weight loss becomes constant first from higher to lower ash content biomass.

In DTG diagrams, three peaks and two non-changing zones can be identified. Firstly, there is a rapid moisture release occurring until 105°C. Then, between temperatures 100–170°C, the biomass absorbs energy without undergoing decomposition. After reaching the ignition temperature around 170°C, a small peak is observed in all samples, mainly associated with hemicellulose devolatilization. This is followed by the maximum decomposition peak attributed to tar and char formation through cellulose consumption [59,60]. Subsequently, another zone with no changes is observed around 350–450°C, during which the biomass absorbs energy. Finally, another peak is observed, corresponding to the combustion of char and tar.

The curves presented in Figures 3 and 4 are consistent with studies conducted by other authors who have thermally characterized agricultural and industrial residues from sugarcane [35,61]. These studies demonstrate mass loss profiles that indicate the progressive and differentiated degradation of biomass constituents, over the same temperature ranges. This includes an initial event related to the evaporation of water and low molecular weight components, followed by a distinct shoulder representing hemicellulose decomposition, positioned just prior to the maximum degradation peak associated with cellulose degradation. The final event marks the combustion of the formed char.

### 3.5. Combustion performance analysis

Table 7 shows that the highest peak temperatures Tp were obtained for bagasse and pith at 320°C with the highest decomposition rates. The lowest peak temperatures were obtained for the two GHR species at 310 and 314°C with lower decomposition rates. In general, the peak temperatures obtained for the different residues are very similar to each other, with a maximum difference of 10°C between them. The high decomposition rates observed in bagasse and pith are directly correlated with their volatile matter content and biochemical composition. Previous research has demonstrated that hemicellulose and cellulose, both characterized by their high oxygen content, decompose into small molecules in the form of highly volatile gases [62,63]. The degradation of these components induces a structural transformation in the biomass, increasing its porosity, thereby enhancing the combustion performance [64]. This finding aligns with other studies that have shown higher combustion rates and greater reactivity in biomasses with similar characteristics to bagasse and pith, in terms of their volatile matter and oxygen content, as well as their biochemical composition [65,66].

**Table 7.** Biomass reactivity for combustion.

	Temperatures (°C)			Rate (%/min)			
Biomass feedstock	T_i_	T_p_	T_b_	R_max_	R_av_	D*10^4^ (%min^−1^°C^2^)	S*10^6^ (%^2^min^−2^°C^3^)
GHR1	168	314	526	8.87	1.65	16.81	9.85
GHR2	172	310	516	6.37	1.37	11.95	5.73
SB	196	320	496	18.32	1.97	29.20	18.91
SP	198	320	494	17.38	2.08	27.43	18.71

Reactivity is typically calculated using mathematical relations that involve maximum and average decomposition rates, R_max_ and R_av_, as well as characteristic temperatures. First, ignition temperature (Ti) is defined as the temperature at which the decomposition rate changes after moisture release [67]. Second, the peak temperature (Tp) is where the maximum decomposition rate (R_max_) occurs, and finally, burnout temperature (Tb) is the point where no further decomposition occurs [68]. While many thermogravimetric analysis studies include these temperatures, there is no standardized methodology for calculating Ti and Tb. In this study, Ti was determined as the inflection point following moisture release. This calculation was done using the second derivative of the percentage weight with respect to temperature. Additionally, Tb was characterized as the temperature at which the magnitude of the combustion rate falls again below 1% per minute after combustion.

Ignition and combustion indexes enable the classification of the studied biomass from less to more reactive. SB and SP yielded higher S and D indexes, approximately twice as much as those observed for GHR samples. Biomass reactivity is influenced by physical characteristics such as particle shape and plant structure, as well as elemental composition, O/C and H/C molar ratios, and biochemical composition [69]

In general, the characteristic temperatures for biomass are lower compared to coal [70]. Meanwhile, decomposition rates are higher for coal which leads to higher values for both the ignition index D and the combustion index S. Bagasse characteristic temperatures Ti and Tb are similar to those reported by [71], but the ignition temperature is significantly lower than the 285°C reported in that study. This difference arises from notable composition disparities. While both samples exhibit similar volatile matter content (around 79%), in the referenced study the sample has a 10% higher carbon content and an 8% lower oxygen content, which implies volatile matter with less reactive oxygen compounds. Consequently, this results in a higher ignition temperature compared to the present work. Also, as mentioned before, ignition temperature definition is not standardized. In the study of Chen et al. [70], the use of a higher heating rate (20°C/min) shifts the DTG to the right, hence increasing characteristic temperatures.

The characteristic temperatures are lower for GHRs compared to SB and SP. This discrepancy can be attributed to the structural differences in the plant sources. While industrial residues, such as SB and SP, primarily consist of fibers from the stems post-juice extraction, GHR primarily comprises leaves. The observed differences in reactivity come from these structural distinctions.

Stems, which contribute to SB and SP, possess greater hardness due to the arrangement of multiple layers of robust, thick-walled cells bound together by lignin. This lignin, being less reactive compared to hemicellulose and cellulose, the primary polymeric compounds in leaves, accounts for the variation in reactivity [28,72,73]. Although GHR exhibits higher lignin content than SB and SP, the lignin in the stems, which eventually form SB and SP residues, is more stable. This stability arises because, after juice extraction, the soluble and less stable lignin is removed due to the acidic nature of the sugarcane juice [74].

In terms of ignition and combustion indexes, D and S, it is evident that GHRs exhibit lower reactivity compared to SB and SP. This discrepancy primarily comes from variations in volatile matter content, oxygen content, and fixed carbon, as these factors influence decomposition rates and characteristic temperatures. The differential thermograms of SB and SP closely resemble each other. This is because SP is a residue derived from SB, resulting in very similar characteristics between the two. For GHR1, proximate analysis indicates that the volatile matter and ash contents are very similar to those of SB. However, due to the higher O/C ratio in SB, it experiences a higher maximum decomposition peak. This is attributed to the presence of more oxygen compounds on the surface, making SB more reactive in combustion. Conversely, GHR2 possesses the lowest volatile matter content and the highest ash content among the biomasses analyzed. Consequently, it undergoes lower devolatilization compared to the other biomass samples.

The thermal behavior and composition of sugarcane residues, as discussed earlier, play a pivotal role in their potential utilization for bioenergy production. This study reveals a pivotal factor influencing the utilization of sugarcane agricultural residues for bioenergy production – the weather conditions during the collection period. This insight holds significant implications for the development of effective utilization strategies. Furthermore, the findings demonstrate that despite certain compositional differences, the overall thermal behavior of sugarcane residues remains consistent. This consistency opens up exciting possibilities for the potential integration of agricultural residues as complementary fuels, including the option of blended formulations alongside industrial residues. Through the application of pre-treatment processes to enhance their purity and reactivity, agricultural residues can potentially play a pivotal role in bioenergy production. This research not only sheds light on the seasonal variations and biochemical composition of sugarcane residues but also highlights the viability of specific strategies, such as biomass fractionation, for enhancing their utilization in the bioenergy sector. These findings mark a significant contribution to the field and open avenues for more sustainable and efficient bioenergy production practices.

## 4. Conclusions

This study assessed the thermochemical behavior of agricultural and industrial sugarcane residues. Results revealed comparable composition, except for agricultural residue GHR2, collected in rainy season, which showed elevated ash content, potentially leading to operational challenges. Agricultural residues displayed lower O/C ratios and higher lignin content than industrial residues, resulting in lower decomposition rates during combustion. In contrast, industrial residues exhibited higher combustion stability. Despite lower decomposition peaks in agricultural residues, combustion occurred over a similar temperature range compared to industrial residues. This provides insights for optimizing sugarcane residue utilization for bioenergy, emphasizing the significance of seasonal variations and biochemical composition.

## 5. Recommendations for future work

In consideration of potential future studies, several key areas warrant exploration. Firstly, conducting diverse experiments with various sugarcane varieties and employing different collection methods, across various seasons and locations, would yield more representative and reliable data regarding their properties and potential for bioenergy applications. Secondly, investigating the efficacy and feasibility of techniques such as washing and fractionation to reduce the ash content of agricultural residues collected during the rainy season represents important research to deepen. Furthermore, a comprehensive kinetic analysis of the Thermogravimetric Analysis (TGA) data is recommended. This analysis should aim to obtain crucial kinetic parameters, including activation energy, pre-exponential factor, reaction order, or frequency factor for each type of residue. Additionally, it would be valuable to conduct an economic analysis to assess the investment costs associated with collecting, preparing, and utilizing these residues for combustion, as well as exploring the potential for formulating blends with industrial residues. Finally, extending the scope of the study to explore other thermochemical conversion processes, such as pyrolysis or gasification for sugarcane residues, and subsequently comparing their performance with combustion in terms of energy yield, quality, efficiency, emissions, and other relevant factors, could offer valuable insights. These recommendations provide potential directions for further exploration, building upon the foundations laid by the current study.

## Data Availability

The data that support the findings of this study are available from the corresponding author, Juan Guerrero, upon reasonable request.

## Abbreviations

GHR: green harvesting residues
TGA: thermogravimetric analysis
SB: sugarcane bagasse
SP: sugarcane pith
R_max_: maximum mass combustion rate
R_av_: average mass combustion rate
T_i_: ignition temperature
T_p_: peak temperature
T_b_: burnout temperature
D: ignition index
S: combustion index
HHV: high heating value
LHV: lower heating value
db: dry basis
dafb: dry ash-free basis
T_in_: initial temperature
C_p,gas_: heat capacity for produced gas
T_adiab_: adiabatic temperature
f: mass ratio fuel/air
DTG: differential thermogravimetric analysis
